# Supplementary material for: Eating the brain - A multidisciplinary study provides new insights into the mechanisms underlying the cytopathogenicity of Naegleria fowleri
Source: PLoS Pathog. 2025 Mar 17;21(3):e1012995. doi: 10.1371/journal.ppat.1012995 (PMC11964265; doi:10.1371/journal.ppat.1012995)
Supplement: S1 Fig — Ingested parts of HT1080 cells are visible in Naegleria vacuoles. Nuclei were labeled with Hoechst 33342 (blue). Scale bar=10 µm. (PDF) [file ppat.1012995.s002.pdf]

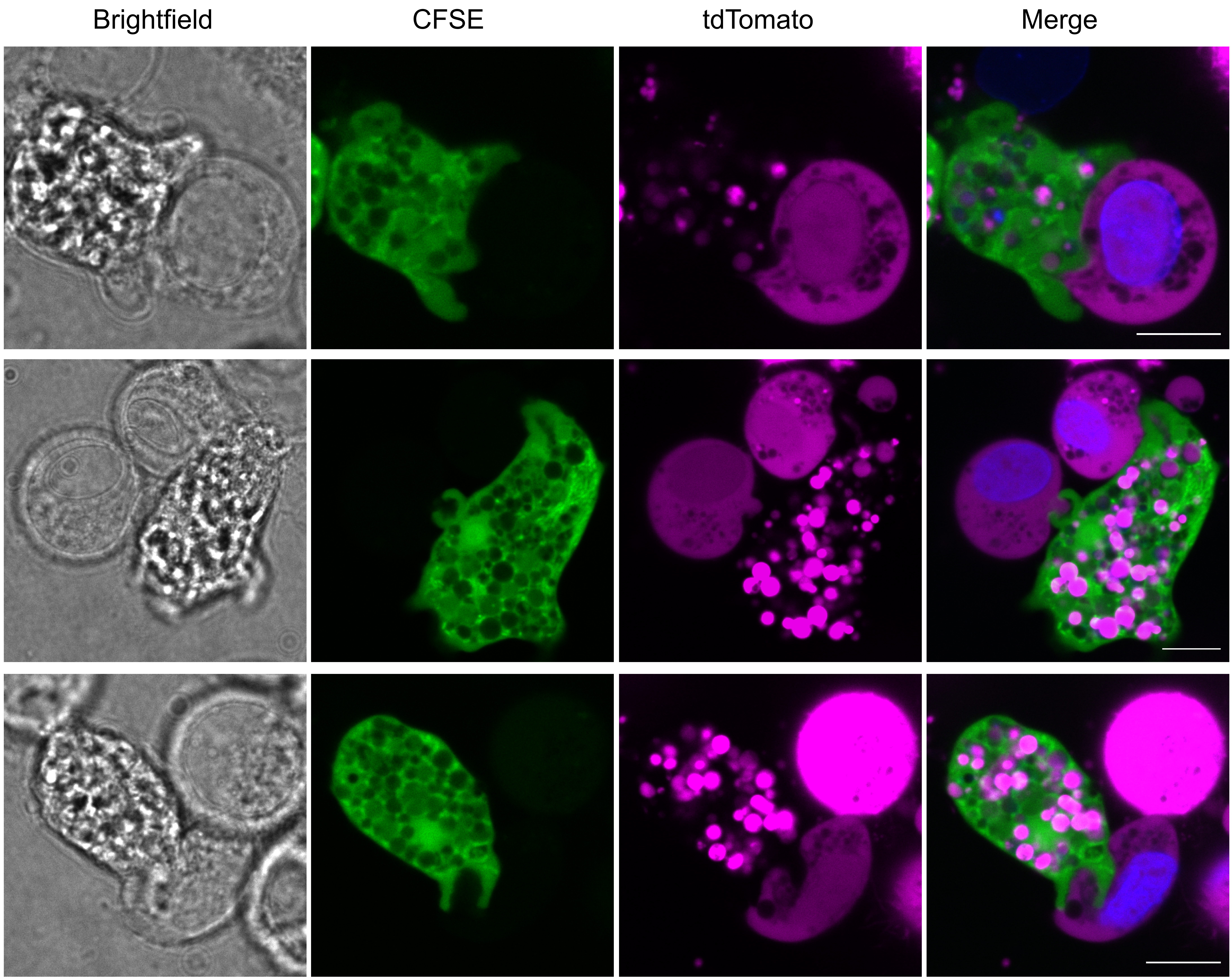

S1 Fig: Live imaging of CFSE-labeled *Naegleria fowleri* (green) and HT1080 fibrosarcoma celss with tdTomato protein in cytosol (magenta) co-cultures showing the process of *Naegleria* adhesion to human cells. Ingested parts of HT1080 cells are visible in *Naegleria* vacuoles. Nuclei were labeled with Hoechst 33342 (blue). Scale bar = 10 µm.
