# Supplementary material for: Eating the brain - A multidisciplinary study provides new insights into the mechanisms underlying the cytopathogenicity of Naegleria fowleri
Source: PLoS Pathog. 2025 Mar 17;21(3):e1012995. doi: 10.1371/journal.ppat.1012995 (PMC11964265; doi:10.1371/journal.ppat.1012995)
Supplement: S2 Fig — (A) Flow cytograms of CFSE-labeled N. fowleri incubated with selected concentrations of inhibitors for 3 hours, showing no changes in the gated amoeba population. (B) Flow cytograms of HT1080 tdTomato cells incubated with selected concentrations of inhibitors for 3 hours showing no changes in the gated mammalian cell population. (C) Flow cytograms of CFSE-labeled N. fowleri preincubated with inhibitors in co-culture with HT1080 tdTomato cells. Cytopathogenicity is indicated by the number of Naegleria with ingested cell parts, represented by the red fluorescence of the tdTomato (orange) after 3 hours of co-culture. Preincubation with DMSO was used as a control. (PDF) [file ppat.1012995.s003.pdf]

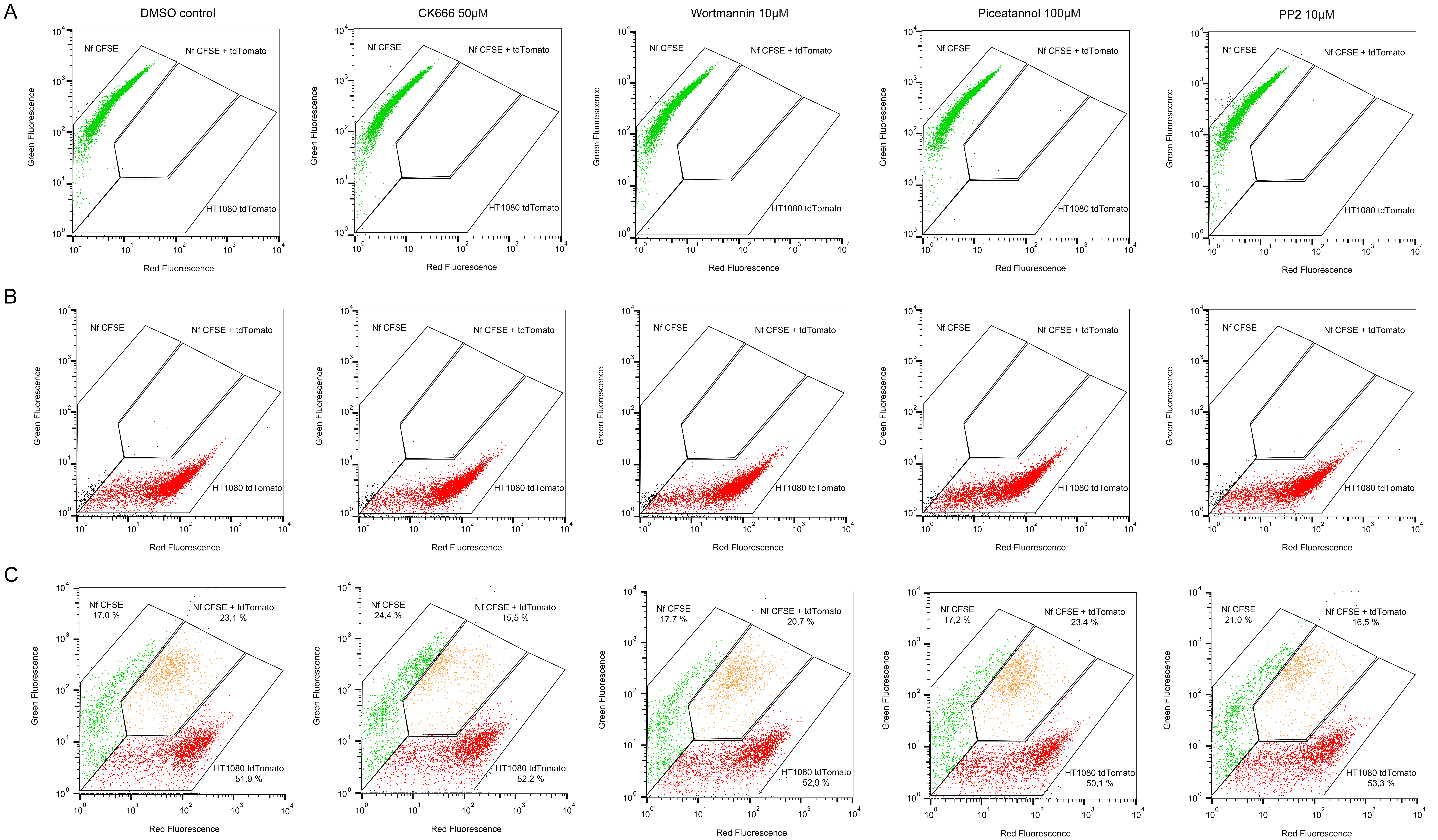

S2 Fig: Representative flow cytograms of CFSE-labeled *Naegleria fowleri* (green), HT1080 cells expressing tdTomato (red) and their co-culture after pre-incubation with different inhibitors (CK-666, wortmannin, piceatannol and PP2). (A) Flow cytograms of CFSE-labeled *N. fowleri* incubated with selected concentrations of inhibitors for 3 hours, showing no changes in the gated amoeba population. (B) Flow cytograms of HT1080 tdTomato cells incubated with selected concentrations of inhibitors for 3 hours showing no changes in the gated mammalian cell population. (C) Flow cytograms of CFSE-labeled *N. fowleri* preincubated with inhibitors in co-culture with HT1080 tdTomato cells. Cytopathogenicity is indicated by the number of *Naegleria* with ingested cell parts, represented by the red fluorescence of the tdTomato (orange) after 3 hours of co-culture. Preincubation with DMSO was used as a control.
