# Supplementary material for: Eating the brain - A multidisciplinary study provides new insights into the mechanisms underlying the cytopathogenicity of Naegleria fowleri
Source: PLoS Pathog. 2025 Mar 17;21(3):e1012995. doi: 10.1371/journal.ppat.1012995 (PMC11964265; doi:10.1371/journal.ppat.1012995)
Supplement: S3 Fig — Lysozyme domain is shown in purple; peptidoglycan binding domain is shown in yellow, and disordered regions are shown in gray. (PDF) [file ppat.1012995.s004.pdf]

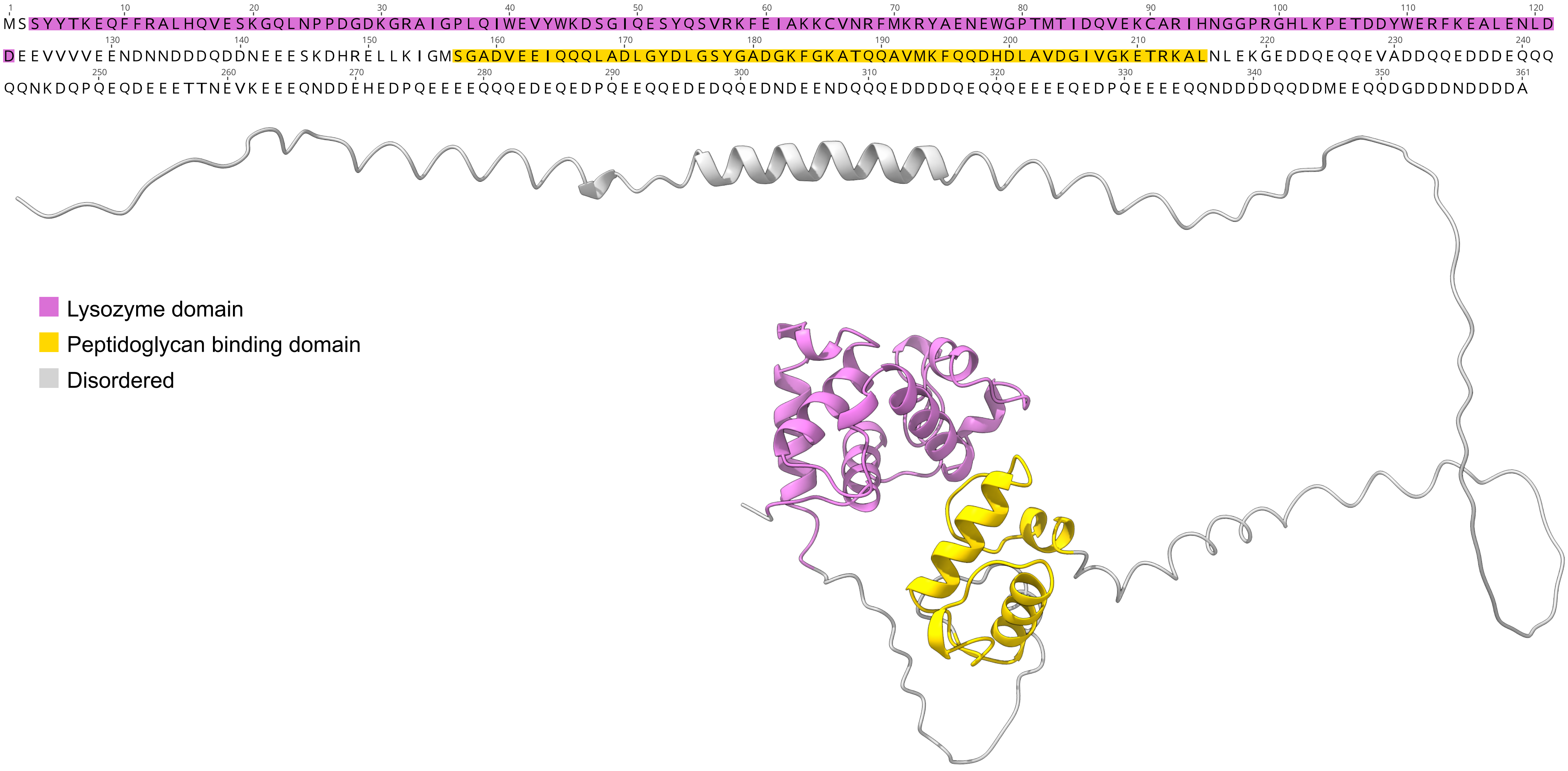

S3 Fig: Protein sequence and structure of *Naegleria fowleri* lysozyme (A0A6A5BDE0) predicted by Alphafold. Lysozyme domain is shown in purple; peptidoglycan binding domain is shown in yellow, and disordered regions are shown in gray.
