# Supplementary material for: Eating the brain - A multidisciplinary study provides new insights into the mechanisms underlying the cytopathogenicity of Naegleria fowleri
Source: PLoS Pathog. 2025 Mar 17;21(3):e1012995. doi: 10.1371/journal.ppat.1012995 (PMC11964265; doi:10.1371/journal.ppat.1012995)
Supplement: S4 Fig — (PDF) [file ppat.1012995.s005.pdf]

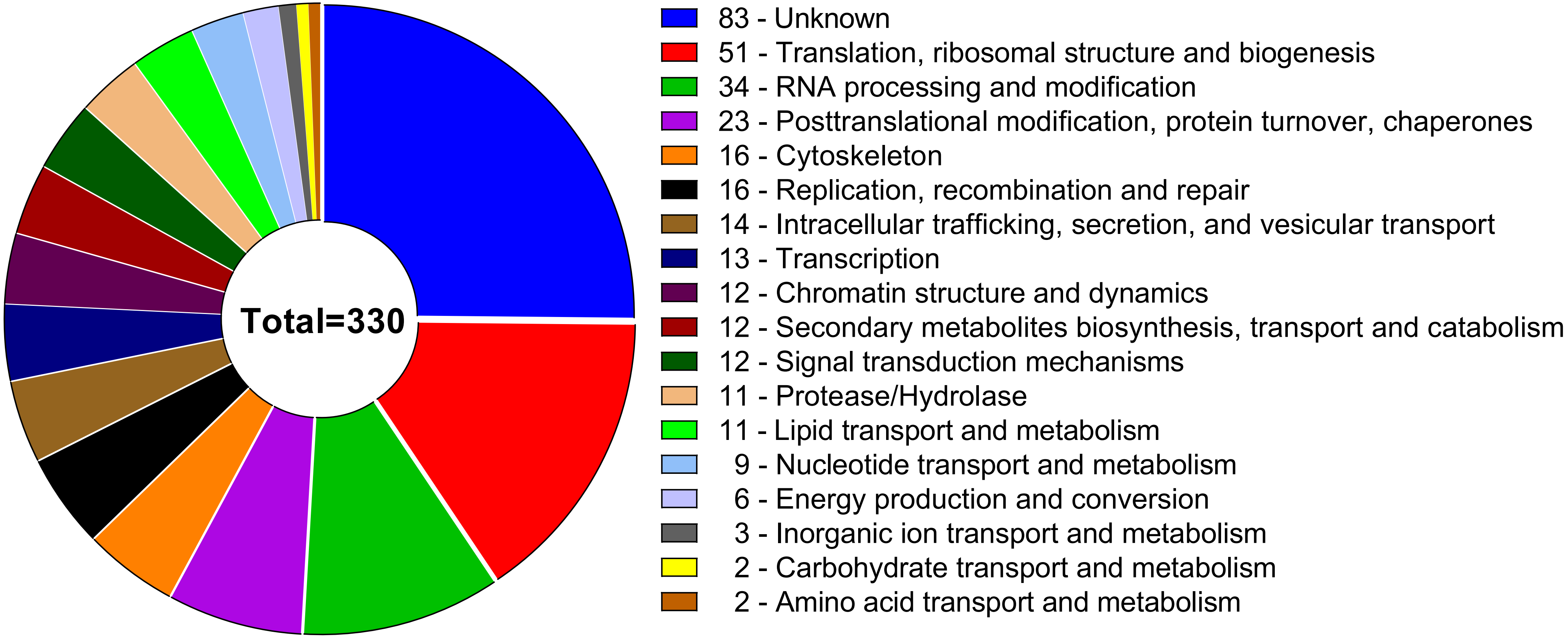

S4 Fig: Proteins upregulated in Naegleria fowleri isolated from mice brains in comparative proteomics sorted into functional categories using HHpred annotation.
