# Supplementary material for: Eating the brain - A multidisciplinary study provides new insights into the mechanisms underlying the cytopathogenicity of Naegleria fowleri
Source: PLoS Pathog. 2025 Mar 17;21(3):e1012995. doi: 10.1371/journal.ppat.1012995 (PMC11964265; doi:10.1371/journal.ppat.1012995)
Supplement: S5 Fig — Cystatin domain is shown in purple with cystatin motif in blue and signal presequence is shown in yellow. (PDF) [file ppat.1012995.s006.pdf]

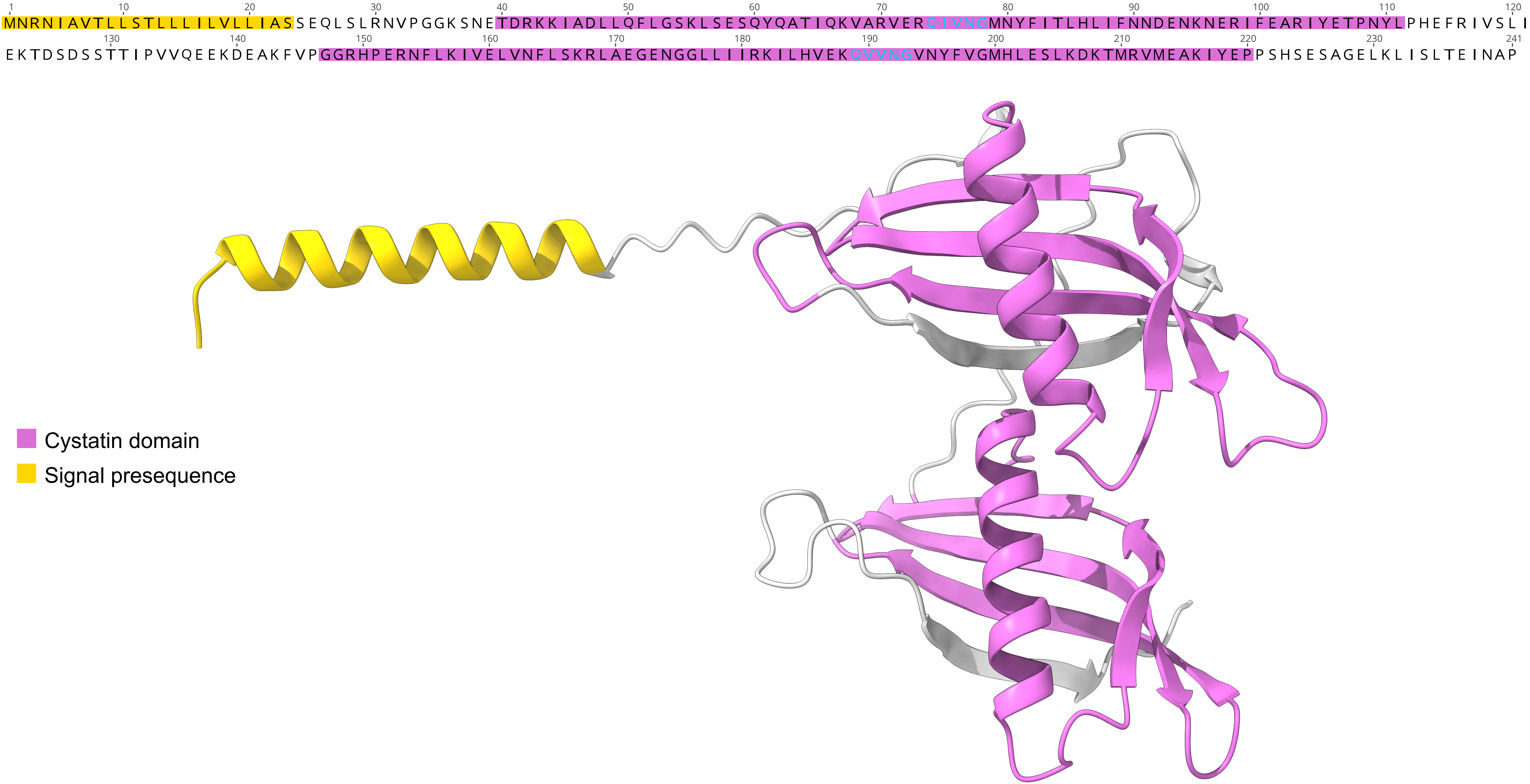

S5 Fig: Protein sequence and structure of *Naegleria fowleri* cystatin (A0A6A5BXL1) predicted by Alphafold. Cystatin domain is shown in purple with cystatin motif in blue and signal presequence is shown in yellow.
