# Supplementary material for: Eating the brain - A multidisciplinary study provides new insights into the mechanisms underlying the cytopathogenicity of Naegleria fowleri
Source: PLoS Pathog. 2025 Mar 17;21(3):e1012995. doi: 10.1371/journal.ppat.1012995 (PMC11964265; doi:10.1371/journal.ppat.1012995)
Supplement: S6 Fig — The molecular weights calculated for each significant peak in the chromatograms are shown within tables. Molecular weights were calculated from calibration curves of the Gel Filtration Standards (Bio-Rad, USA) and for hemerythrin from Gel Filtration LMW Calibration Kit (Cytiva, USA). The peak of the standards along with their molecular weight is listed at the top of the chromatographs. The inset in each chromatograph shows SDS-PAGE of the recombinant protein. (PDF) [file ppat.1012995.s007.pdf]

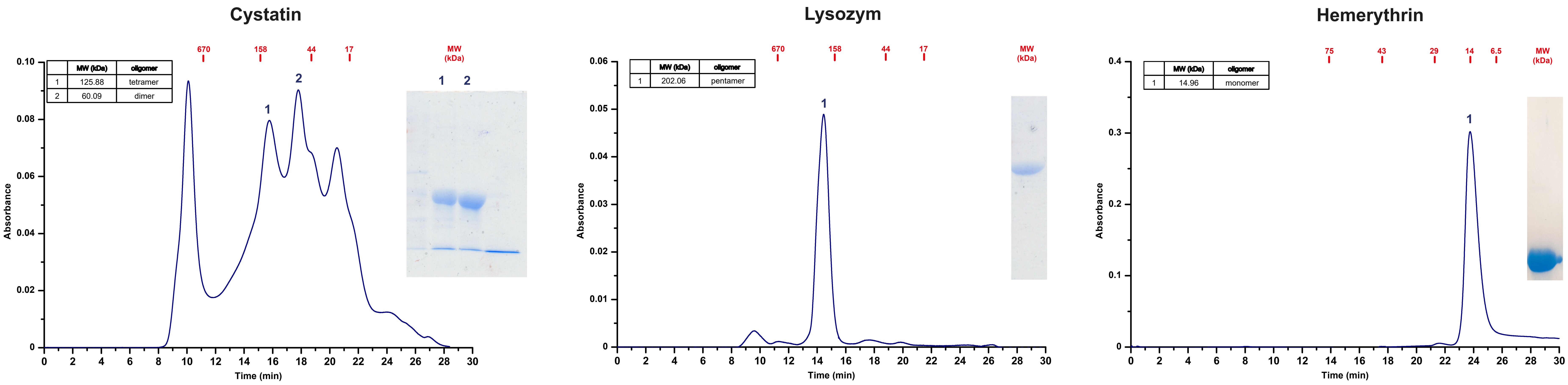

S6 Fig: Chromatograms from size-exclusion chromatography of recombinant *Naegleria fowleri* cystatin, lysozym and hemerythrin. The molecular weights calculated for each significant peak in the chromatograms are shown within tables. Molecular weights were calculated from calibration curves of the Gel Filtration Standards (Bio-Rad, USA) and for hemerythrin from Gel Filtration LMW Calibration Kit (Cytiva, USA). The peak of the standards along with their molecular weight is listed at the top of the chromatographs. The inset in each chromatograph shows SDS-PAGE of the recombinant protein.
