# Supplementary material for: Eating the brain - A multidisciplinary study provides new insights into the mechanisms underlying the cytopathogenicity of Naegleria fowleri
Source: PLoS Pathog. 2025 Mar 17;21(3):e1012995. doi: 10.1371/journal.ppat.1012995 (PMC11964265; doi:10.1371/journal.ppat.1012995)
Supplement: S7 Fig — (A) Enzymatic activity of recombinant Naegleria fowleri lysozyme (blue line) and chicken egg white lysozyme (red line). A decrease in absorbance at 450 nm over time is associated with lysozyme enzymatic activity. The black line represents a blank sample without enzyme addition. (B) Control of protein loading for the immunoblot of N. fowleri lysozyme in Fig 4, stained by Ponceau S. ax.: total lysate from axenically cultured N. fowleri, g-: N. fowleri co-cultured with Klebsiella aerogenes, g+: N. fowleri co-cultured with Micrococcus lysodeikticus, fib.: N. fowleri co-cultured with HT1080 fibrosarcoma cells. (PDF) [file ppat.1012995.s008.pdf]

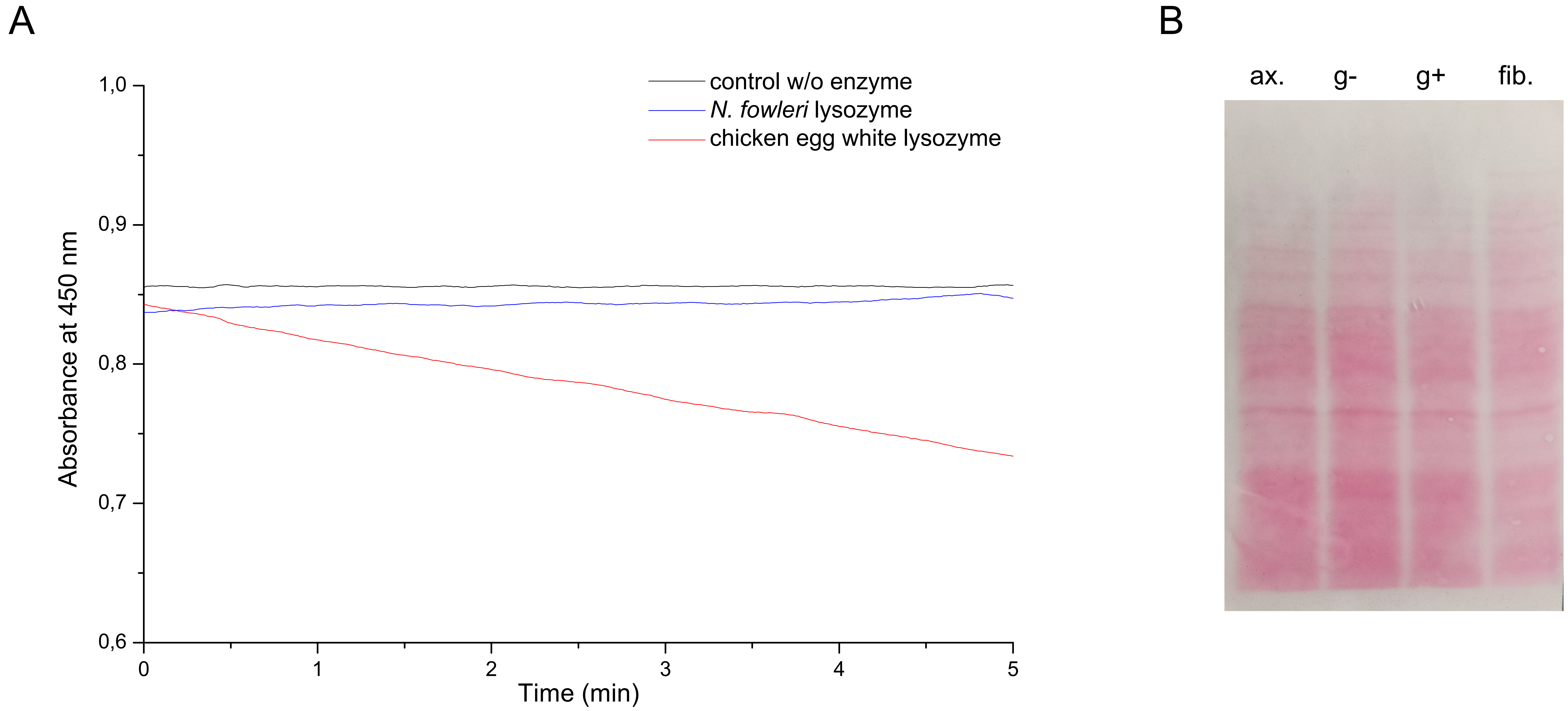

S7 Fig: Enzymatic activity of lysozyme and control of protein loading. (A) Enzymatic activity of recombinant *N. fowleri* lysozyme (blue line) and chicken egg white lysozyme (red line). A decrease in absorbance at 450 nm over time is associated with lysozyme enzymatic activity. The black line represents a blank sample without enzyme addition. (B) Control of protein loading for the immunoblot of *Naegleria fowleri* lysozyme in Fig. 4, stained by Ponceau S. ax.: total lysate from axenically cultured *N. fowleri*, g-: *N. fowleri* co-cultured with *Klebsiella aerogenes*, g+: *N. fowleri* co-cultured with *Micrococcus lysodeikticus*, fib.: *N. fowleri* co-cultured with HT1080 fibrosarcoma cells.
