# Supplementary material for: Eating the brain - A multidisciplinary study provides new insights into the mechanisms underlying the cytopathogenicity of Naegleria fowleri
Source: PLoS Pathog. 2025 Mar 17;21(3):e1012995. doi: 10.1371/journal.ppat.1012995 (PMC11964265; doi:10.1371/journal.ppat.1012995)
Supplement: S9 Fig — Cytopathogenicity is indicated by the number of Naegleria with ingested cell parts, represented by the red fluorescence of the tdTomato (orange) after 1 and 3 hours of co-culture. (PDF) [file ppat.1012995.s010.pdf]

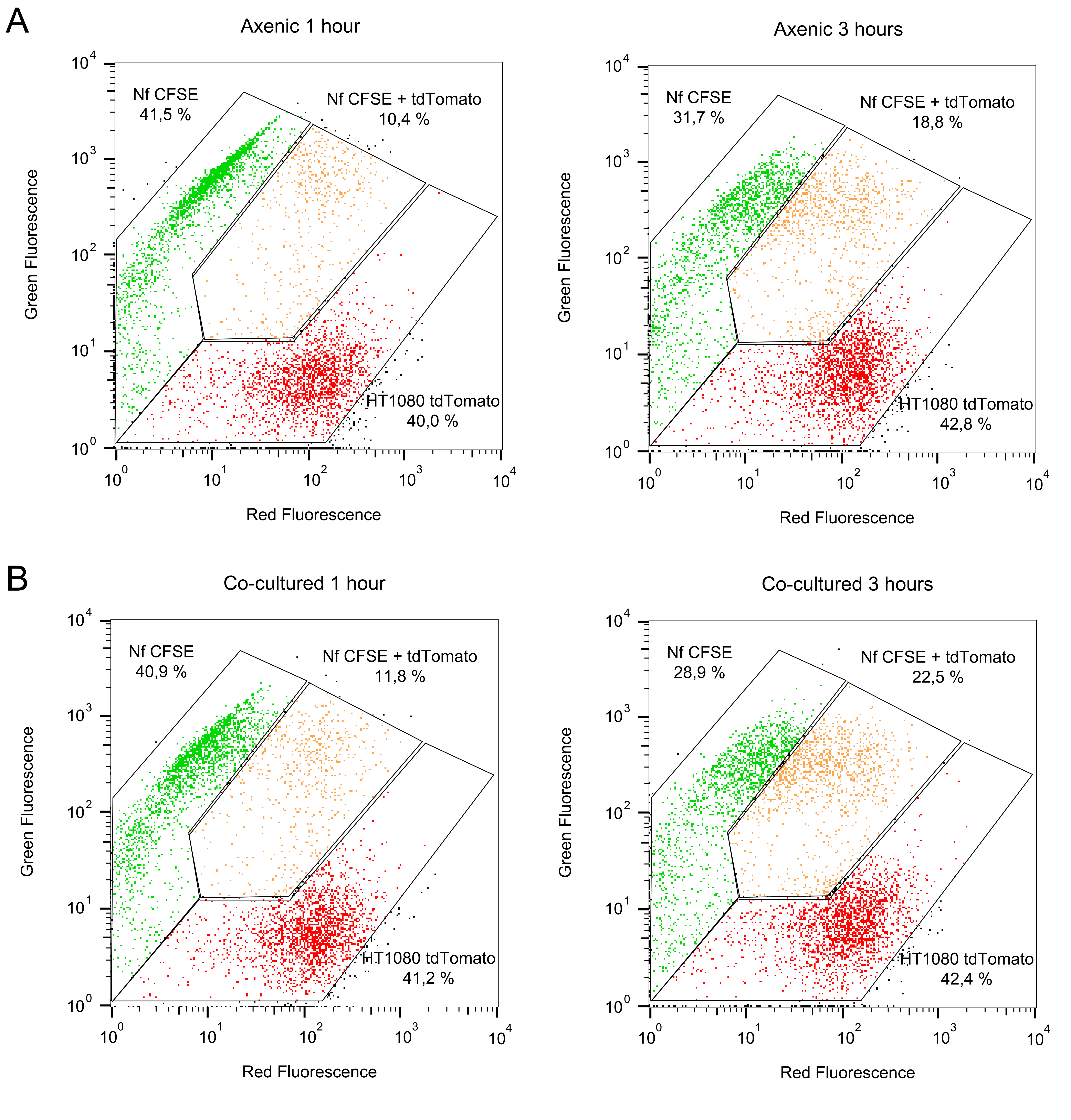

S9 Fig: Representative flow cytograms of CFSE labelled (A) axenic *Naegleria fowleri* and (B) long-term co-cultured *Naegleria fowleri* (green) in co-culture with HT1080 cells expressing tdTomato (red). Cytopathogenicity is indicated by the number of *Naegleria* with ingested cell parts, represented by the red fluorescence of the tdTomato (orange) after 1 and 3 hours of co-culture.
