# Supplementary material for: Eating the brain - A multidisciplinary study provides new insights into the mechanisms underlying the cytopathogenicity of Naegleria fowleri
Source: PLoS Pathog. 2025 Mar 17;21(3):e1012995. doi: 10.1371/journal.ppat.1012995 (PMC11964265; doi:10.1371/journal.ppat.1012995)
Supplement: S10 Fig — (A) Graph of doubling time of axenically cultured N. fowleri (axenic) and long-term co-cultured N. fowleri (co-cultured) in host cell-free medium. (B) Graphs showing changes in the percentage of CellTrace Far Red-labeled axenic or long-term co-cultured N. fowleri (Nf CelltraceFR) with ingested either cytosolic eGFP or membrane-targeted eGFP-CAAX from HT1080 cells after 3 hours of co-incubation as measured by flow cytometry. (** p-value<0.01) (C) Live imaging of CellTrace Far Red-labeled N. fowleri (magenta) and HT1080 fibrosarcoma cell with eGFP-CAAX (green) co-culture showing the ingestion of human cell plasma membrane by amoebae. Scale bar=10 µm. (PDF) [file ppat.1012995.s011.pdf]

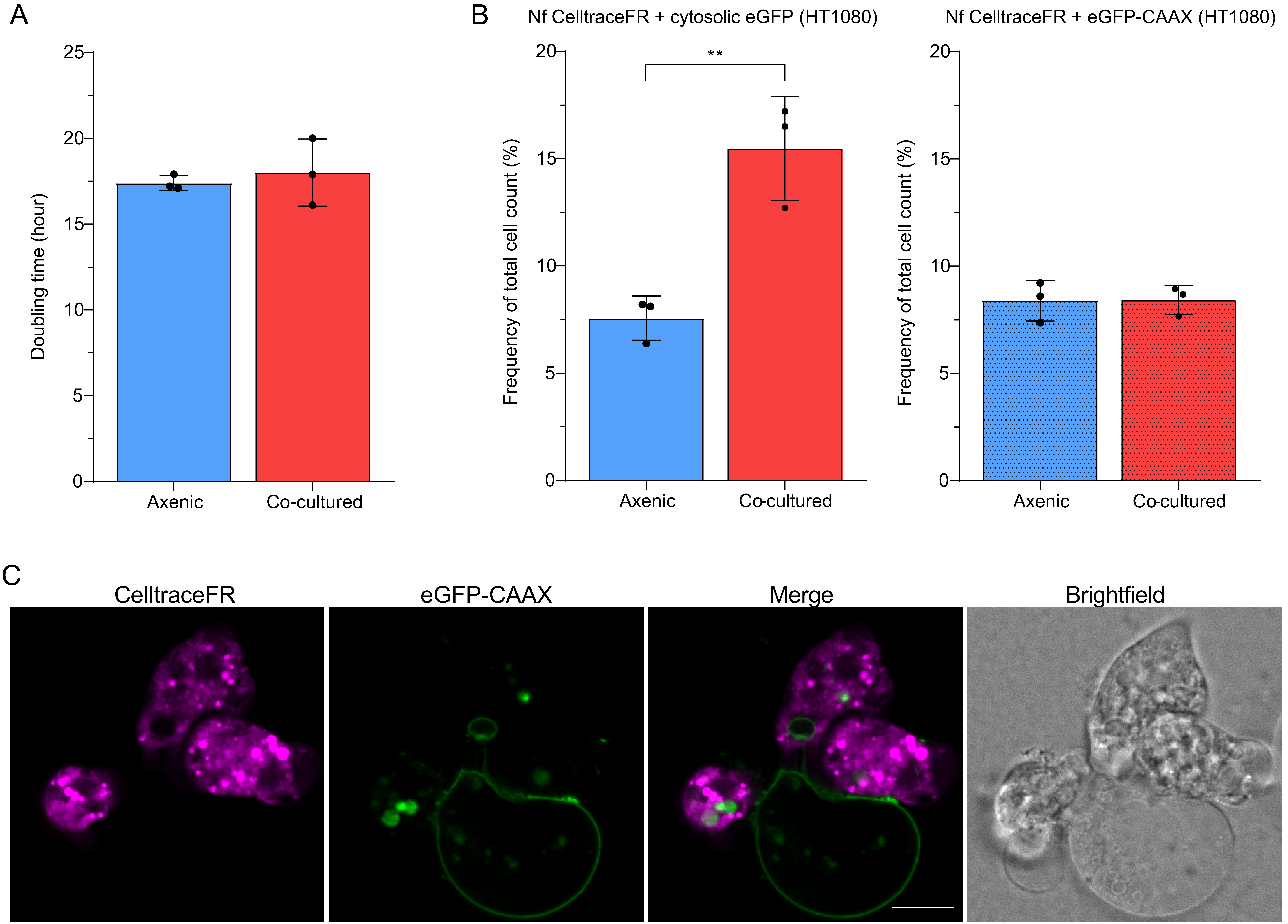

S10 Fig: Long-term co-culture of *Naegleria fowleri* with mammalian cells affects amoeba cytopathogenicity but has no effect on its proliferation rate and trogocytosis. (A) Graph of doubling time of axenically cultured *N. fowleri* (axenic) and long-term co-cultured *N. fowleri* (co-cultured) in host cell-free medium. (B) Graphs showing changes in the percentage of CellTrace Far Red-labeled axenic or long-term co-cultured *N. fowleri* (Nf CelltraceFR) with ingested either cytosolic eGFP or membrane-targeted eGFP-CAAX from HT1080 cells after 3 hours of co-incubation as measured by flow cytometry. (** p-value<0.01) (C) Live imaging of CellTrace Far Red-labeled *N. fowleri* (magenta) and HT1080 fibrosarcoma cell with eGFP-CAAX (green) co-culture showing the ingestion of human cell plasma membrane by amoebae. Scale bar = 10 μm.
