# Supplementary material for: Eating the brain - A multidisciplinary study provides new insights into the mechanisms underlying the cytopathogenicity of Naegleria fowleri
Source: PLoS Pathog. 2025 Mar 17;21(3):e1012995. doi: 10.1371/journal.ppat.1012995 (PMC11964265; doi:10.1371/journal.ppat.1012995)
Supplement: S11 Fig — These maps illustrate significantly different phenotypes of both cultivation conditions based on the major spectral components representing the cytoplasm and nucleus detectable in most of the measured cells (with some exceptions when the confocally limited optical section missed the nucleus) or ingested cells and lipid droplets detected in Naegleria cells isolated from the brain. (PDF) [file ppat.1012995.s012.pdf]

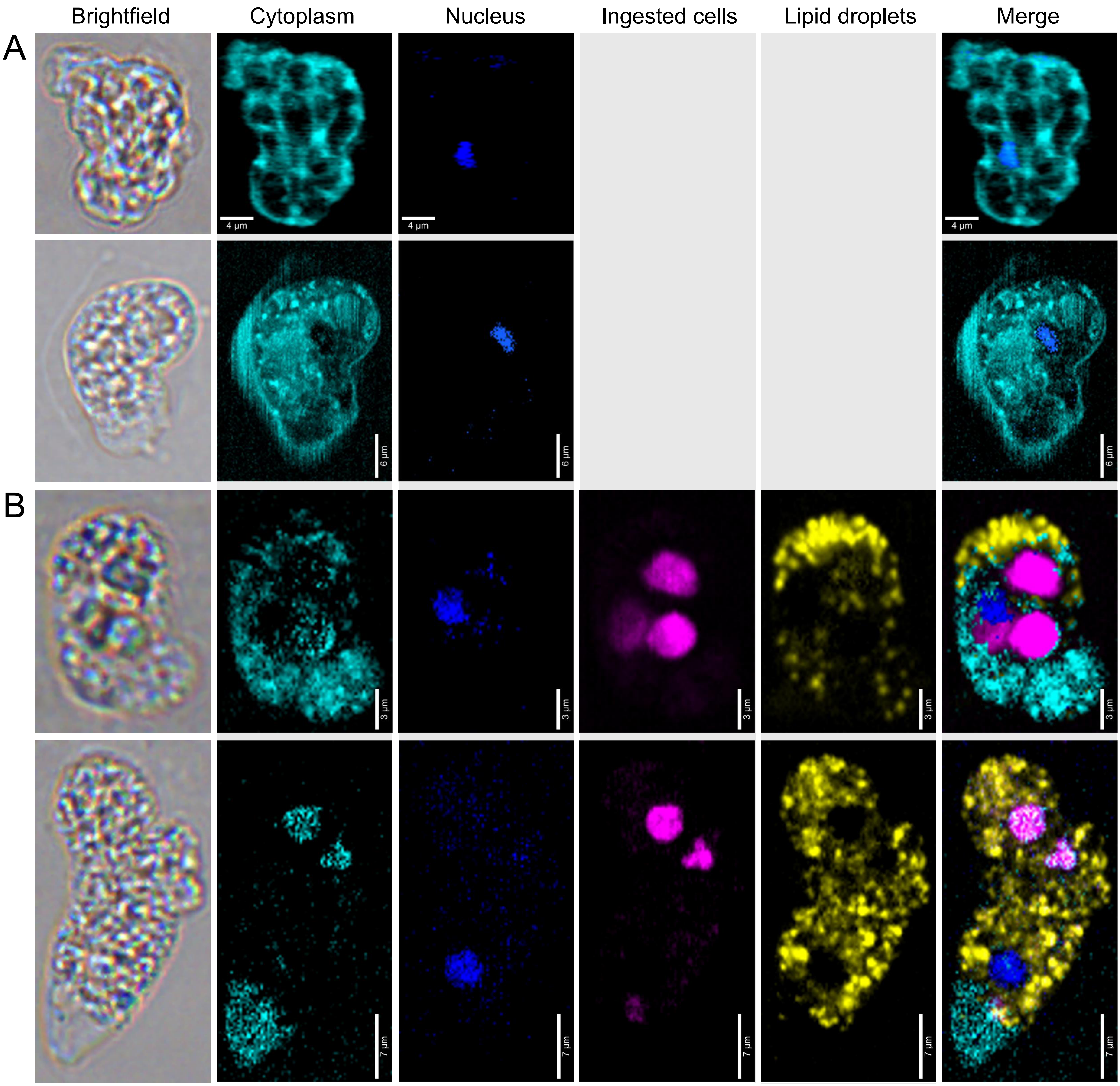

S11 Fig: Raman chemical maps of *N. fowleri* cultivated axenically (A) and isolated from a mouse brain (B) illustrate significantly different phenotypes of both cultivation conditions based on the major spectral components representing the cytoplasm and nucleus or ingested cells and lipid droplets detected in *Naegleria* cells isolated from the brain.
