# Supplementary material for: Eating the brain - A multidisciplinary study provides new insights into the mechanisms underlying the cytopathogenicity of Naegleria fowleri
Source: PLoS Pathog. 2025 Mar 17;21(3):e1012995. doi: 10.1371/journal.ppat.1012995 (PMC11964265; doi:10.1371/journal.ppat.1012995)
Supplement: S7 Table — (DOCX) [file ppat.1012995.s019.docx]

**S7 Table**

| **Sample preparation** | Tissues were homogenized and lysed by boiling at 95°C for 10 min in 100 mM TEAB (Triethylammonium bicarbonate) containing 2% SDC (sodium deoxycholate), 40 mM chloroacetamide, 10 mM TCEP (Tris(2-carboxyethyl)phosphine) and further sonicated (Bandelin Sonoplus Mini 20, MS 1.5). Protein concentration was determined using BCA protein assay kit (Thermo Fisher Scientific) and 30 µg of protein per sample was used for MS sample preparation.  Samples were further processed using SP3 beads according to (1). Briefly, 5 µl of SP3 beads was added to 30 µg of protein in lysis buffer and filled to 50 µl with 100 mM TEAB. Protein binding was induced by addition of ethanol to 60% (vol./vol.) final concentration. Samples were mixed and incubated for 5 min at RT. After binding, the tubes were placed into magnetic rack and the unbound supernatant was discarded. Beads were subsequently washed two times with 180 µl of 80% ethanol. After washing, samples were digested with trypsin (trypsin/protein ration 1/30) reconstituted in 100 mM TEAB at 37°C overnight. After digestion samples were acidified with TFA to 1% final concentration and peptides were desalted using in-house made stage tips packed with C18 disks (Empore) according to (2). |
| --- | --- |
| **Enzyme specificity** | Trypsin/P with max 2 miscleavages and minimal and maximal peptide length 7 and 25 amino acids respectively |
| **Trap column** | C18 PepMap100, 5 μm particle size, 300 μm x 5 mm (Thermo Fisher Scientific) 4 min, 18 μl/min; loading buffer (2% acetonitrile, 0.1% trifluoroacetic acid, water); elution with mobile phase gradient from 4% to 35% in 120 min; 1 ug of sample was loaded on the column. |
| **LC-MS column** | Nano Reversed phase column (EASY-Spray column, 50 cm x 75 µm ID, PepMap C18, 2 µm particles, 100 Å pore size) |
| **Mobile phases** | Mobile phase buffer A (water and 0.1% formic acid); mobile phase B (acetonitrile and 0.1% formic acid) |
| **Peptides analysis** | Gas-phase ions were analyzed by a Thermo Orbitrap Fusion (Q-OT- qIT, Thermo Fisher Scientific); survey scans from 350 to 1400 m/z in orbitrap; resolution – 120 K (200 m/z); target ion count - 5 x 10^5^; tandem MS - 1,5 Th quadrupol; HCD fragmentation collision energy - 30; rapid scan MS analysis in the ion trap; MS2 ion count target - 10^4^; max injection time - 35 ms; precursors with charge state 2–6 were sampled for MS2; dynamic exclusion duration - 45 s with a 10 ppm tolerance; top speed mode; 2 s cycles. |
| **Peak list, search engine** | All data were processed by MaxQuant 2.0.3.0 with built-in search engine Andromeda, which was also used for peak list generation, peptides assignment to proteins and data normalization (according to (3)); MS1 maximum peak intensity was used for quantification. |
| **Sequence database** | For the data evaluation were used following protein databases: *Mus musculus* (downloaded from uniprot.org on January 2022 containing 17 059 entries), human (downloaded from uniprot.org on July 2021 containing 20 612 entries), *Klebsiella aerogenes* (downloaded from uniprot.org on November 2021 containing 4 909 entries) and *Naegleria fowleri* (downloaded from AmoebaDB on December 2021, containing 13 925 entries). Whole-cell proteome samples were searched against the *Mus musculus*, Human, *Klebsiella aerogenes* and *Naegleria fowleri* databases, and samples from the analysis of both secretomes were searched against the Human and *Naegleria fowleri* databases. |
| **Data analysis** | Analysis was performed using Perseus 1.6.15.0 software (4); statistical relevance was estimated using students t-test with Benjamini-Hochberg’s correction (FDR = 0.05) |
| **Fixed modifications** | Carbamidomethyl on cysteine (+57.02146) |
| **Variable modifications** | Oxidation (M), Acetyl (Protein N-term) |
| **Mass tolerance for precursor ions** | 20 ppm for first search (before recalibration) and 4.5 ppm for main search (after recalibration) |
| **Mass tolerance for fragment ions** | 0.5 Da |
| **False Discovery Rates at Peptide and Protein levels** | 1 % at both peptide and protein level |

1. Hughes CS, Moggridge S, Müller T, Sorensen PH, Morin GB, Krijgsveld J. Single-pot, solid-phase-enhanced sample preparation for proteomics experiments. *Nat Protoc*. 2019;14(1):68-85

2. Rappsilber J, Mann M, Ishihama Y. Protocol for micro-purification, enrichment, pre-fractionation and storage of peptides for proteomics using StageTips. *Nat Protoc*. 2007;2(8):1896-1906.

3. Cox J, Hein MY, Luber CA, Paron I, Nagaraj N, Mann M. Accurate proteome-wide label-free quantification by delayed normalization and maximal peptide ratio extraction, termed MaxLFQ. *Mol Cell Proteomics*. 2014;13(9):2513-2526.

4. Tyanova S, Temu T, Sinitcyn P, et al. The Perseus computational platform for comprehensive analysis of (prote)omics data. *Nat Methods*. 2016;13(9):731-740.
