# Supplementary material for: Eating the brain - A multidisciplinary study provides new insights into the mechanisms underlying the cytopathogenicity of Naegleria fowleri
Source: PLoS Pathog. 2025 Mar 17;21(3):e1012995. doi: 10.1371/journal.ppat.1012995 (PMC11964265; doi:10.1371/journal.ppat.1012995)
Supplement: S8 Fig — Protein alignment of the hemerythrins with the iron-binding amino acids highlighted. (PDF) [file ppat.1012995.s009.pdf]

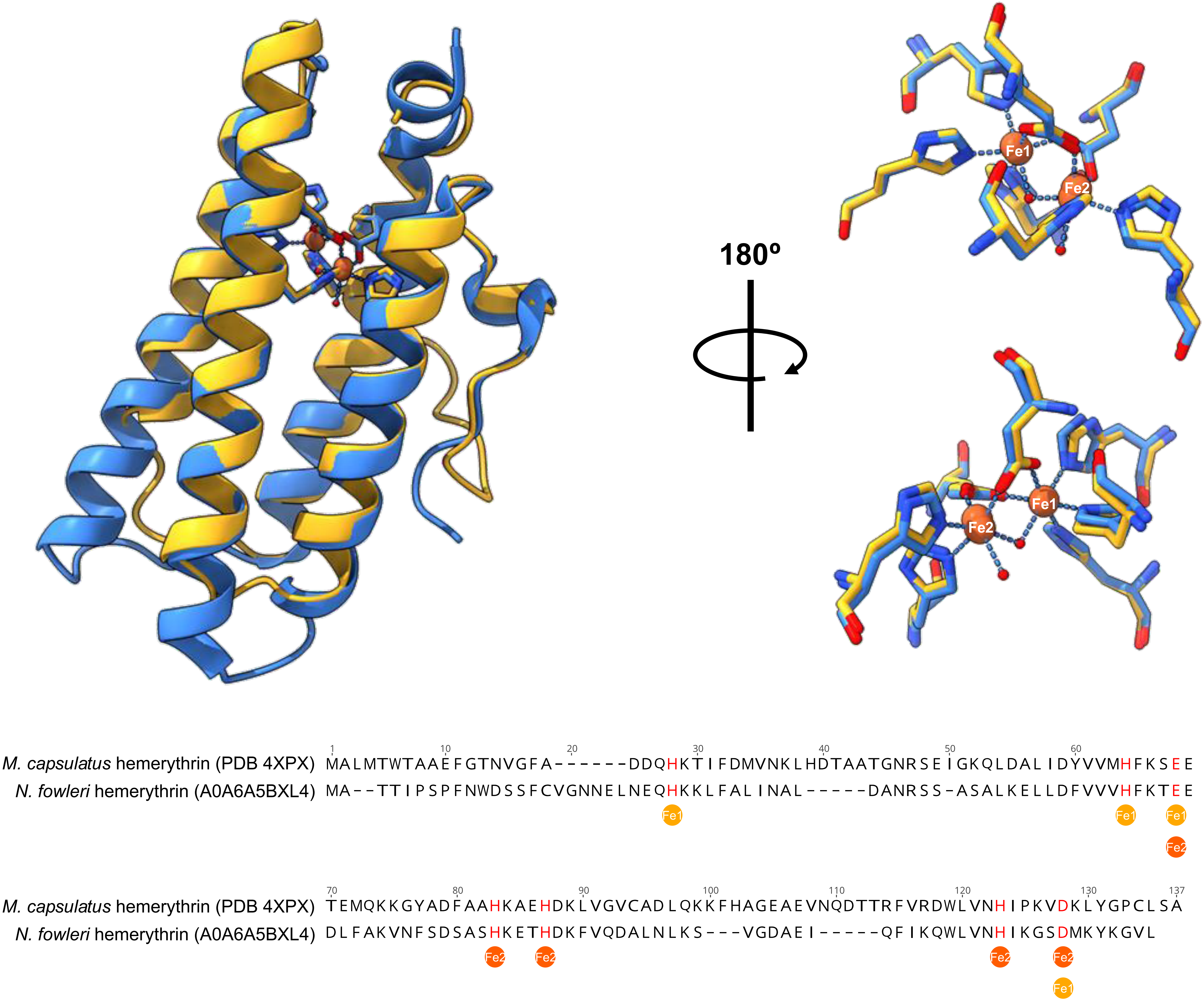

S8 Fig: Structural alignment of the *Naegleria fowleri* hemerythrin (A0A6A5BXL4, yellow) predicted by Alphafold and the crystallographic structure of the *Methylococcus capsulatus* hemerythrin (PDB 4XPX, blue). Protein alignment of the hemerythrins with the iron-binding amino acids highlighted.
