# Supplementary material for: Eating the brain - A multidisciplinary study provides new insights into the mechanisms underlying the cytopathogenicity of Naegleria fowleri
Source: PLoS Pathog. 2025 Mar 17;21(3):e1012995. doi: 10.1371/journal.ppat.1012995 (PMC11964265; doi:10.1371/journal.ppat.1012995)
Supplement: Attachment — Submitted filename: Response to Reviewers.docx [file ppat.1012995.s021.docx]

**Reviewers' Comments:**

Reviewer's Responses to Questions

**Part I - Summary**
Please use this section to discuss strengths/weaknesses of study, novelty/significance, general execution and scholarship.

Reviewer #1: The manuscript by Malych et al. investigates how Naegleria fowleri amoebae interact with mammalian host cells. This is an understudied topic, and more work in the area is needed to understand this deadly pathogen and ultimately devise new therapeutics. The authors first show that actin is important for consumption of human cells using flow cytometry. They then compare proteomes of amoebae cultured in different conditions, and identify and characterize proteins that are upregulated in mouse-passaged amoebae. They go on to analyze secreted proteins, and end with Raman Microspectroscopy comparing axenic to mouse-passaged amoebae. The variety of techniques used is quite impressive, the flow of the paper is logical, and the findings could influence the manner in which Naegleria’s cell biology is studied (in co-culture vs mouse, see point 1). This work would be of broad interest to researchers studying eukaryotic pathogens, or comparing cell-based and mouse models. This work would also be very useful to the Naegleria community. While we appreciate the amount of work that went into this study, we have a few issues that we believe should be addressed.

Reviewer #2: The goal of this study by Malych et al. is to identify factors at the protein level that uniquely empower virulence within the Naegleria fowleri compared with other Naegleria species. The authors use an in vitro co-culture system with human fibrosarcoma feeder cells and N. fowleri of different passage histories (axenic, mouse, etc) to attempt to define proteins that could be involved in contact-dependent and independent cytopathogenicity. Several complementary in vitro, in silico, and proteomic approaches were used, with some outcomes being more conclusive than others. In the absence of in vivo tests, the relevance of these findings are ultimately difficult to know, but they nonetheless add to our overall understanding of Naegleria biology. There are several areas where the authors could/should improve this manuscript.

Reviewer #3: This manuscript effectively explores various potential virulence factors that the amoeba can utilize under different conditions.

I have few important questions to address. Furthermore, I want to highlight that the introduction offers a comprehensive and ordered overview of the history of some important virulence factors previously described in the amoeba.

1. Is there a specific reason you chose to use this fibrosarcoma cell line?

We chose this cell line because it is an established model human cell line in biomedical research. It is easy to culture in a reproducible manner. In addition, through collaborators, we have access to differently labeled strains, e.g. membrane-labeled cells, which were used in this resubmission (new S10 Fig).

2. you mention in lines 85 to 88 that "we investigated the mechanism of contact-dependent cytopathogenicity using inhibitors of actin and several protein kinases related to cellular processes such as motility and adhesion". The question is:
Why were only actin and kinase inhibitors chosen to assess the contact-dependent mechanism? What led to the decision to consider solely actin as contact-dependent? Do you consider that some of analyzed enzymes play a role as contact-dependent mechanisms?

Our goal was not to elucidate the contact-dependent mechanism in detail, and the actin inhibitors were used more as a proof of concept for our co-culture system. But we admit that we were a bit too bold in the text and therefore we have removed this part of the sentence “To further elucidate role of trogocytosis in host cell killing by *N. fowleri* and”.

3. In lines 139 you mention that " We further focused on the analysis of the 18 upregulated proteins that were shared between the long-term fibrosarcoma co-culture and brain-isolated amoebae as these are likely to be involved in the
interaction with the host"
What reasons could explain why proteins previously reported as contact-dependent for the amoeba, such as integrin-like proteins, membrane protein (Mp2CL5), or Nfa1, do not appear on this particular list (18 upregulated proteins)?

While comparative proteomics is a powerful tool for identifying proteins involved in cellular responses to the environment, lack of inducibility does not mean that proteins are not involved in these processes. Their basal expression may be sufficient and their post-translational modification or relocation in the cell may lead to their involvement in host interactions. We added the sentence: “In our proteomic dataset, the expression of Nfa1 is not induced in the long-term fibrosarcoma co-culture and brain-isolated amoebae, nor are other suggested virulence factors such as integrin-like proteins (8) and Mp2CL5 (33).” into the manuscript to highlight these proteins.

4. In line 152 you mention that "This lysozyme (here after named as Nf lysozyme) contains two domains: a lysozyme and a peptidoglycan binding domain (protein sequence and structure prediction is depicted in S3 Fig).

5. In line 172 tha athors mention that "the expression of Nfa1 did not significantly change between conditions, but the paralog of this protein, hemerythrin (A0A6A5BXL4), was identified only in brain-isolated N. fowleri"

Why did the expression of Nfa1 remain largely unchanged despite the direct interaction with the brain? The evidence has been supported that this protein serves as a significant adhesion factor in vitro. What implications does this have for this result?

As mentioned above, the lack of inducibility does not mean that the protein could not be involved in the processes. The level of Nfa1 in amoebae could be constant and the protein would be relocated from the cytosol to the food cups to help the cell with adhesion only after the stimuli.

How would you explain and through which domain of the enzyme (lysozyme) the union with some host protein would be carried out (contact-dependent manner), and if you could only suggest some host receptor according to this type of enzyme?

Unfortunately, a large part of Nf lysozyme consists of disordered region, which makes any bioinformatic structural predictions of potential interaction partners very difficult. We have tried to predict the secondary structure of Nf lysozyme using different models (Alphafold2, Alphafold3, Chai-1), but none of them could resolve the disordered region (as can be seen in the attachment). Therefore, in the future we would like to perform e.g. co-immunoprecipitation experiments in search for potential interaction partners of this protein.

6. In line 189-192 the authors report Nf cystatins structures. Do you think that these enzymatic structures could be participating in contact-dependent binding to a receptor on the host cells?

This is a possibility, but cystatins are a group of proteins with very diverse biological functions. Therefore, without an effective knockdown system in *Naegleria*, we dare not predict the function.

7. In lines 203-205 the authors mention that " Immunoblots showed an increase in Nf lysozyme protein levels after long-term co-culture with both gram-positive bacteria and fibrosarcoma cells, suggesting its involvement in the interaction with the prey".

It would likely be more appropriate to state that the protein showed an increase in antibody recognition intensity, suggesting a more active involvement of this protein.

Thank you for the suggestion. The sentence has been rewritten as: “Immunoblots showed an increase in the intensity of antibody recognition of Nf lysozyme, suggesting an active involvement of this protein in the interaction with some bacteria and with mammalian cells, which could have an impact on the infection of the host.” to highlight the active involvement of this protein.

When the authors refer to "prey," are they talking about bacteria specifically, or do they mean all the cells they interacted with, including mammalian cells? Additionally, which band corresponds to the protein with an approximate molecular weight of 25 kDa (fig 4 A), and what proteins does it represent?

In this context, the term prey is used to describe all cells with which the amoebae interact. We believe that the 25 kDa protein may represent another lysozyme that is specific towards Gram-positive bacteria and therefore is not upregulated in amoebae co-cultured with mammalian cells.

I am unsure if the image quality and resolution are the final version, but they are not the best.

We apologize for the low quality of the images, which was caused during the upload to the system. The final images will be delivered in the required quality.

**Part II – Major Issues: Key Experiments Required for Acceptance**
Please use this section to detail the key new experiments or modifications of existing experiments that should be absolutely required to validate study conclusions.

Generally, there should be no more than 3 such required experiments or major modifications for a "Major Revision" recommendation. If more than 3 experiments are necessary to validate the study conclusions, then you are encouraged to recommend "Reject".

Reviewer #1: 1. The finding that the proteome of mouse passaged amoebae is so different from long-term co-cultured amoebae seems potentially very impactful. Does this throw into question the usefulness of experiments using co-culture systems? We think this could be a major take-away from this work that is worth discussing. While this major point does not involve experiments, I think addressing this point would strengthen the impact of the manuscript.

While the co-culture system is very important for ease of measurement of cytopathogenicity and better reproducibility, we are aware that the mouse model provides more information because, unlike the co-culture system, *Naegleria* must deal with e.g. oxidative stress and extracellular matrix. We acknowledge that this aspect was underestimated in the manuscript and therefore we added a bioinformatic analysis of proteins upregulated in the brain (new S4 Fig and S2 Table). We also added this new paragraph into the results: “As mentioned above, 330 proteins are upregulated in the brain. Among the proteins with predicted function by HHpred (Supplementary Table, Fig.), 16 are cytoskeletal proteins related to migration in tissues, 11 are proteases/hydrolases probably important for virulence, and finally 11 are lipid metabolism proteins, indicating that the molecules are a source of energy for the amoebae in the brain, as we show in further results.”

2. I have some hesitations about the flow cytometry data. For figures 1A, 1C, and 5D, how were the gates for red or green fluorescence determined? It would be appropriate to run amoebae alone (+/- inhibitors) and human cells alone (subject to the same treatments and dyes but without amoebae) to determine the borders of the gates. The imaging shown in Movie S1 is beautiful, but if I’m not mistaken, there is detectable CFSE background in the human cells, which could result in human cells that have taken up some amount of residual CFSE being counted as amoeba that have consumed mammalian cells. Further, the inhibitors used could lead to changes in either the amoebae or mammalian cells that may impact the fluorescence (e.g. if it results in any cell death or leakage). I think it is worth being a bit cautious with this gating, because if I understand the methods correctly, the amoebae and drugs were mixed 1:1 with human cells, which would mean the human cells are also subjected to a 3 hour incubation with these inhibitors, which could change their biology, especially at some of the concentrations used (also see minor point 4).

Gates for red and green fluorescence were first determined on labeled amoebae and mammalian cells alone and verified prior to each flow cytometry co-culture experiment. We understand the concern about inhibitor concentrations since they are not washed away and are only diluted 2-fold after mixing amoebae with mammalian cells. Therefore, we prepared a flow cytometry experiment in which we incubated *Naegleria* and HT1080 cells individually for 3 hours with the inhibitor concentrations used in the amoeba pre-incubation of our co-culture experiments. This new result shows the stability of our gating, as it did not change for either the amoeba or the mammalian cell population after the 3-hour incubation. We have added these results to S2 Fig and hope that it better clarifies our flow cytometry results. Regarding the CFSE background, while we labelled the amoebae for most of the experiments, we used unlabelled amoebae in the holotomography shown in movie S1. For better clarity we have added to the description of the S1 movie and methodology that we used unlabeled *Naegleria* for this experiment. Nevertheless, after labeling the amoebae, the remainder of the dye that was not covalently bound to proteins inside the cell was washed away.

Reviewer #2: The authors show that inhibition of actin networks impairs feeding behavior, as has been reported in N. gruberi. The authors should explain how know that actin is used by Naegleria in general for feeding advances our understanding of why N. fowleri is apparently the only species of Naegleria that is pathogenic.

We do not believe that feeding behavior plays a role in the ability of *N. fowleri* to infect humans and therefore did not address this aspect in the manuscript. In fact, we know from our own experiments that *N. gruberi* feeds on human cells just as efficiently as *N. fowleri*. This intriguing question of *N. fowleri* ability to parasitize humans, which is probably triggered by multiple factors such as thermostability or the ability to resist the immune system, is something we will continue to investigate in the future.

For experiments using mouse passaged amoeba, the authors should state how long after mouse infection amoeba harvested and how long after harvest from mouse are amoeba used.

To ensure a high and stable infection rate, we passaged the amoebae through the mice twice prior to the experimental infections. Infected mice were euthanized after 5 days of infection, and amoebae were isolated directly from their brains into growth medium and used for further experiments approximately 2 hours after isolation. The process was better clarified in the Methodology in Mouse Infection: “Amoeba cells were isolated directly from the brains of euthanized mice after 5-days of infection and placed into growth medium in culture flasks at 37°C until a sufficient number of parasites had migrated from the brain (≈2 hours). These amoebae were used directly for further experiments.”

In Figure 1C—are inhibitors washed away from amoeba or are they maintained in inhibitor solution while plated with feeders cells ? Methods don’t specifically address this.

Inhibitor concentrations are diluted only 2-fold after mixing amoebae with mammalian cells. We understand the concern that this might change the biology of the human cells and consequently the gating. Therefore, we prepared a flow cytometry experiment in which we incubated *Naegleria* and HT1080 cells individually for 3 hours with the inhibitor concentrations and determined the potential changes in gated populations. The results are shown in the new S2 Fig and show no significant changes in the gated populations.

Can the authors functionally demonstrate differences in production of protease/proteases from using different N. fowleri conditions with feeder cells/axenic etc conditions?

Although we would like to perform such a functional experiment, we do not believe it is experimentally feasible. In our case, the significant induction of protease secretion required contact with the host cell, which results in the destruction of host cells and therefore the release of host proteins, including proteases, which would interfere with the measurement. Therefore, we chose a proteomic approach that allowed us to filter out the host proteins, leaving only the amoeba proteins.

Naegleria have not evolved to feed on mammalian tissues while facing an mammalian immune system, they are accidental pathogens. With that in mind, it would be useful to frame the production of these factors less through the lens of attacking the host, but rather upregulating feeding mechanisms that are appropriate for the food source. Blocking cysteine protease activity and scavenging free radicals would conceivably allow N. fowleri to live longer in a host, but what “normal” purpose might these serve. Why would amoeba be using these in their natural environment compared with other species of N. fowleri?

This is a fair point, but we are not suggesting that the virulence mechanisms are only for survival in the host and specific to *Naegleria fowleri*. In a complex environment with other organisms, these factors could help *N. fowleri* to fight its predators or to process some prey. For example, in the article by Uzuka et al. (2019), they show that free-living amoebae may have to cope with the photosynthetic oxidative stress of photosynthetic prey. We believe that the advantage of *N. fowleri* over other *Naegleria* that gives it the ability to infect potential hosts is mainly based on the ability to survive at higher temperatures, escape the immune system, and possibly mediated by the lysozyme discussed in our manuscript, which is *N. fowleri* specific. However, without establishing a knockout line for this protein, we cannot be 100% certain of its role in infection.

Uzuka, A., Kobayashi, Y., Onuma, R., Hirooka, S., Kanesaki, Y., Yoshikawa, H., Fujiwara, T., & Miyagishima, S. Y. (2019). Responses of unicellular predators to cope with the phototoxicity of photosynthetic prey. Nature communications, 10(1), 5606. https://doi.org/10.1038/s41467-019-13568-6

The results presented here and in prior literature indicate that some N. fowleri proteins have the ability to inhibit some proteases from different mammalian hosts. How do the authors interpret lines 206-209 (i.e what does this really mean?) There is discussion of cystatins, but not how the authors envision these highly variably pairings of activity/function could result in a functional impacts in vivo---especially when there are many proteases that would likely not be inhibited?

The ability of a canonical inhibitor (here the Nf cystatin) to block the activity of a particular peptidase depends on the structure of the peptidase active site and its amino acid composition on one hand, and on the structure of the inhibitor on the other hand. In the case of cysteine cathepsins (Merops Clan CA), the S2 subsite of the active site has been shown to be highly important for the specificity towards substrates/inhibitors. Cathepsins B, L and K tested in this study differ in the structures of the S2 binding pocket and this explains why the particular Nf cystatin tested in this study binds to the active sites of just some of the proteases (the in silico modelling – molecular docking – anticipated this result). Eukaryotic organisms in general possess sets of various inhibitors that may partially overlap in their specifities towards proteases while differing in their affinity, but, on the other hand, there is no single universal inhibitor that would act on all proteases. In the case of *N. fowleri*, the situation is similar. For example, another cystatin of the amoeba, fowlerstefin (Thái et al., 2020, https://www.doi.org/10.1186/s13071-020-3909-6) inhibited cathepsin B, which was not the case of the Nf cystatin characterized in this study.

Figure 5: The attempt to disentangle cell destruction via trogocytosis vs that done by cell lysis is an admirable and much needed line of investigation. It’s not clear how the work in Figure 5 answers this.

Thank you for that important point. Indeed, our results show the difference in protein secretion and cytotoxicity between axenic and long-term co-cultured amoebae, but they do not distinguish between cell lysis and trogocytosis because the mammalian cell line used in the flow cytometry experiment contained cytosolic fluorescent protein. In order to focus specifically on trogocytosis, we repeated the flow cytometry experiment using a new HT1080 cell line carrying membrane-targeted GFP (eGFP-CAAX). The results of this experiment are described below.

On line 290, it’s unclear what “…and the percentage of HT1080 cells after 1 and 3 hours of co-culture….” Means. Are words missing?

We apologize for the error in this sentence. The sentence should read: “In our study, we used the flow cytometry method illustrated in Fig 1A and compared the percentage of CFSE-labeled amoebae containing tdTomato fluorescent protein from HT1080 fibrosarcoma cells after 1 and 3 hours of co-culture with either axenically maintained *N. fowleri* or long-term co-cultured *N. fowleri*.” and was corrected in the revised manuscript.

Can this assay (in Figure5) differentially report cells consumed via trogocytosis or lysis? If so, this should be clearly stated. If not, could something similar be done with cells that express either membrane or organelle argeted fluorescent proteins? Or perhaps with physical separation of amoeba and feeders so that trogocytosis is impossible.

Thank you again for this excellent suggestion, which has greatly improved the outcome of our manuscript. To better distinguish between cells consumed by trogocytosis or lysis, we repeated the flow cytometry experiment shown in Figure 5 with a new HT1080 cell line carrying membrane-targeted GFP (eGFP-CAAX). We used CellTrace™ Far Red dye, which works on the same principle as CFSE, to label our amoebae and co-incubated them with either HT1080 cells with cytosolic eGFP or membrane-targeted eGFP-CAAX. This new result shows that long-term co-cultured *Naegleria* ingested more cytosolic eGFP from HT1080 than axenic *Naegleria*, the same as we showed with CFSE-labeled amoebae and tdTomato. However, comparison of their ingestion of membrane-targeted eGFP-CAAX shows no difference, which strongly suggests that there is no change in trogocytosis. These results have been incorporated into the manuscript as S10 Fig and the result of this experiment was reflected in the manuscript.

Along similar lines, do amoeba need to contact feeders in order to upregulate these proteins? Or can they sense their environment and produce cytopathic factors after “smelling” cells nearby.

Since the strong upregulation of proteases in long-term amoebae was observed only after contact with the host, we assume that contact is an important component. Nevertheless, we do not exclude the possibility that they are able to sense prey without contact. In fact, we intend to address this complex issue in our future project with both *Naegleria* and *Acanthamoeba*.

When comparing axenic and mouse passaged (or long term feeder passage), like in Fig5, is the actual proliferation rate different? Or total number of amoeba cells produced when all feeders cells killed/consumed different? It would be useful to understand what functional advantages these more “virulent” cells have, as the differences between groups at 1 and 3hrs are quite modest. Are passaged cells more efficient at killing or more efficient at “growing”?

We admit that we did not consider this possibility. Therefore, for the revised manuscript, we have now measured the proliferation rates of axenic and long-term co-cultured *N. fowleri* in host cell-free medium. We found that there was no change in proliferation and that both axenic and long-term co-cultured *N. fowleri* doubled approximately every 17 hours under our conditions. Results have been added into S10 Fig and have been incorporated into the text.

While I’m admittedly ignorant of Raman microscopy, how can one know that nucleic acids are from mitochondria and not something else?

From our previous microscopy observations, we know that a significant portion of the *N. fowleri* cell is filled with mitochondria (black dots in the attached NHS-ester labeled *N. fowleri* expansion image). These mitochondria have a strong signal from their DNA in the Raman spectra and therefore are visible on the Raman microscope.

Reviewer #3: No Major issue

**Part III – Minor Issues: Editorial and Data Presentation Modifications**
Please use this section for editorial suggestions as well as relatively minor modifications of existing data that would enhance clarity.

Reviewer #1: Minor points:

1. For flow cytometry data, was any initial gating done to remove debris and doublets?

For debris removal, we used the thresholding feature of the Guava easyCyte 8HT flow cytometer, which allows particles with a forward scatter less than the set value not to be displayed. Doublets were removed by gating during the data processing.

2. Was microscopy done on CK-666 treated cells or PP2-treated cells? If this data is available, it would be nice to see in the publication.

We did not perform microscopy on CK-666 treated or PP2 treated co-cultures. From our experience with untreated cells shown in Fig. 1B and S1 Fig., the ingested parts of the host cells were not uniformly distributed among the amoebae and depended on the place in which the microscope objective was focused. Therefore, we consider microscopy to be of little value in the case of amoebae incubated with the inhibitors and we focused only on flow cytometry.

3. I do not think this is necessary for publication, but an experiment the authors may wish to consider to strengthen their claims and distinguish trogocytosis-induced damage from damage done by secreted proteases could be to treat mammalian cells with supernatants from axenic or co-cultured samples and assess damage of mammalian cells (perhaps with AnnexinV staining, or some other dye). This is just a suggestion, and I understand if it is beyond the scope!

Thank you for this suggestion. We absolutely agree that this would be an interesting experiment, and are planning to do it as part of a future project with *Naegleria* and *Acanthamoeba*, where we want to study contactless damage and contactless sensing of these amoebae.

4. Is there any evidence that wortmannin, piceatannol, or PP2 are effective in Naegleria? The amounts used seem quite high relative to the concentrations typically used in mammalian cells (eg. PP2 is often used at <5 nM, but is used here at 10 uM).

The effective concentrations previously described for *Acanthamoeba*, *Entamoeba*, T-cells, and B-cells (Siddiqui et al. 2012, Ralston et al. 2014, Aucher et al. 2008) were used for the concentrations of each inhibitor for *Naegleria*. To be sure that the concentrations are effective, we also tested different ranges of inhibitor concentrations starting at half the concentration used in other systems. In the case of piceatannol and PP2, the concentrations suggested in the articles were ideal (PP2 10 uM, piceatannol 100 uM), as *Naegleria* cells at higher concentrations were visibly damaged when observed under the microscope. In the case of wortmannin, we saw no visible damage, even at concentrations over 100 times higher than those used in other systems. To avoid the non-specific effects of the inhibitor, we chose a concentration of 10 uM.

Siddiqui R, Iqbal J, Maugueret MJ, Khan NA. The role of Src kinase in the biology and pathogenesis of Acanthamoeba castellanii. Parasit Vectors. 2012;5:112. Published 2012 Jun 7. doi:10.1186/1756-3305-5-112

Ralston KS, Solga MD, Mackey-Lawrence NM, Somlata, Bhattacharya A, Petri WA Jr. Trogocytosis by Entamoeba histolytica contributes to cell killing and tissue invasion. Nature. 2014;508(7497):526-530. doi:10.1038/nature13242

Aucher A, Magdeleine E, Joly E, Hudrisier D. Capture of plasma membrane fragments from target cells by trogocytosis requires signaling in T cells but not in B cells. Blood. 2008;111(12):5621-5628. doi:10.1182/blood-2008-01-134155

5. After a 3 hour incubation with CK666, which may also prevent macropinocytosis in axenic cells, did the cells differentiate into flagellates? If so, this may have resulted in less trogocytosis. A sentence in the text to address if this may have occurred would be sufficient.

During the experiment with inhibitors, we monitored all co-cultures microscopically and did not observe the formation of *Naegleria* flagellates. In the new experiment shown in S2 Fig, where we incubated only axenic amoebae with the inhibitors for 3 hours, no flagellates were observed either. We added the sentence: “As controls, *N. fowleri* and HT1080 were incubated individually with the same concentrations of each inhibitor for 3 hours and analyzed by flow cytometer. In addition, amoeba cells were monitored throughout the incubation to ensure that no flagellate cells were formed." in the Methods to address this issue.

6. The discussion could be shortened; some parts feel repetitive with the results.

We have tried to shorten the discussion in the revised manuscript and hope that it is now easier to read.

7. The results section title “Long-term co-culture of N. fowleri with mammalian cells increases protease secretion and trogocytosis” seems misleading. There were more proteases secreted by axenic cells (18) than co-cultured cells (14) in Figure 5A. Did I miss something?

The secretion of proteases is increased only after contact with host cells (Figure 5C). Nevertheless, the title has been changed to: " Long-term co-culture of *N. fowleri* with mammalian cells increases amoeba cytopathogenicity but does not affect trogocytosis " for clarity after the new experiment showing no increase in trogocytosis.

Minor errors/typos the authors may wish to correct:

Line 94 add [a] before significant role

The line has been corrected as suggested.

Line 314 typo “cytoplam”

The line has been corrected to “cytoplasm”.

Line 326 “dis the amoeba”

The line has been corrected to “distinct from the amoeba”.

Line 167 italics for H. sapiens

The line has been corrected as suggested.

Lines 28 says “amoeba cell ingestion” while lines 95-96 say “host cell ingestion.” It may be worth using consistent language or picking a clearer term (like ingestion of host cells by amoebae)

The lines have been rewritten to "ingestion of host cells by amoebae" for better clearance.

Line 105: replace “on” with “the”

The line has been corrected as suggested.

Line 220-221, is this intended to be “UV light”?

Sentence from previous line “This center was further characterized by EPR spectroscopy.” has been deleted for better clarity.

Reviewer #2: Line 314, cytoplasm is misspelled

The line has been corrected as suggested.

On line 326, the word “dis” is written and presumably there is something else missing.

The line has been corrected to “distinct from the amoeba”.

Figures 5D and 5C appear to be swapped (with regard to in text reference)

We apologize for swapping the marking of Figure 5 in the text, which may have caused confusion. This error has been corrected in the text.

Reviewer #3: (No Response)
